# The role of nitric oxide in hypertensive target organ damage in patients without renal impairment: insights from left ventricular global longitudinal strain and albuminuria

**DOI:** 10.1186/s12872-026-05575-5

**Published:** 2026-02-06

**Authors:** Ayca Arslan, Metin Ogun, Dogan Ilis, Inanc Artac, Mahsum Aykal, Muammer Karakayali, Ozturk Demir, Emrah Kaya, Nazlican Buyukyurt, Yavuz Karabag, Ibrahim Rencuzogullari

**Affiliations:** 1 Faculty of Medicine, Department of Cardiology, Kafkas University, Kars, Türkiye; 2 Faculty of Medicine, Department of Biochemistry, Kafkas University, Kars, Türkiye; 3 Department of Internal Medicine, Siverek State Hospital, Sanliurfa, Türkiye; 4 Faculty of Medicine, Department of Cardiology, Kutahya Health Sciences University, Kutahya, Türkiye

**Keywords:** Nitric oxide, Inducible nitric oxide synthase, Left ventricular global longitudinal strain, Albuminuria, Hypertension-mediated organ damage

## Abstract

**Background:**

Hypertension (HT) is a prevalent chronic condition that contributes significantly to morbidity and mortality by leading to target organ damage. Although nitric oxide (NO) underpins endothelial function and has been implicated in several cardiovascular conditions, its association with subclinical hypertension-mediated organ damage remains unclear. This study aimed to investigate the relationship between nitrite/nitrate (NOx)—an indirect index of NO pathways and subclinical myocardial and renal dysfunction in patients with HT.

**Methods:**

This study prospectively screened 433 adult hypertensive patients, of whom 400 were included in a cross-sectional analysis after exclusion of conditions potentially confounding cardiac or renal assessment, including end-stage renal disease requiring dialysis. All participants underwent transthoracic echocardiography to assess ventricle functions using left ventricular global longitudinal strain (LV-GLS) and provided a spot urine sample for the measurement of the urine albumin/creatinine ratio (UACR). In addition to NOx levels, inducible and endothelial nitric oxide synthase levels were evaluated from participants’ blood samples.

**Results:**

NOx levels were significantly associated with reduced LV-GLS and higher UACR values. In multivariate analysis, lower NOx levels were independently associated with impaired LV-GLS (OR: 0.817; 95% CI: 0.783–0.853; *p* < 0.001) and albuminuria (OR: 0.905; 95% CI: 0.877–0.934; *p* < 0.001).

**Conclusions:**

NOx may represent an independent biochemical parameter associated with subclinical myocardial and renal dysfunction in patients with HT.

**Supplementary Information:**

The online version contains supplementary material available at 10.1186/s12872-026-05575-5.

## Background

Hypertension (HT) is a globally prevalent chronic disease and a major public health concern. As a significant risk factor for cardiovascular diseases, uncontrolled and prolonged hypertension can lead to hypertension-mediated organ damage (HMOD), particularly affecting the kidneys and heart [1, 2].

In the cardiovascular system, sustained HT promotes adverse structural remodeling, including left ventricular hypertrophy (LVH), diastolic dysfunction, and ultimately, heart failure [3]. Among echocardiographic parameters, a reduction in left ventricular global longitudinal strain (LV-GLS) is recognized as an early and sensitive marker of subclinical myocardial dysfunction preceding overt LVH and diastolic impairment [4, 5]. Similarly, the kidneys present another major target, where persistent HT may lead to glomerular injury and albuminuria, a key indicator of renal endothelial dysfunction and a strong predictor for both cardiovascular events and the progression of chronic kidney disease [6, 7]. Therefore, early detection of cardiac and renal involvement is essential for improving outcomes in hypertensive patients.

The nitric oxide (NO) radical is a key endothelial-derived mediator of vascular homeostasis, modulating vasodilation, platelet activity, inflammatory signalling, redox balance, and myocardial relaxation. Alterations in NO pathways have been implicated in major cardiovascular diseases—including heart failure, coronary artery disease, atrial fibrillation, and hypertension [8–12]. Because NO is rapidly oxidised in vivo, we assessed NO indirectly from its stable oxidation products—circulating nitrite and nitrate (NOx). However, whether reduced NOx signalling is linked to HMOD—particularly subclinical myocardial deformation and renal microvascular injury—remains insufficiently defined. We therefore hypothesised that lower circulating NOx is independently associated with more impaired LV-GLS and higher urine albumin-to-creatinine ratio (UACR) in patients with HT.

The aim of this study was to evaluate the association between NOx and subclinical HMOD, as assessed by LV-GLS and UACR.

## Methods

### Study design and population

**Fig. 1:**
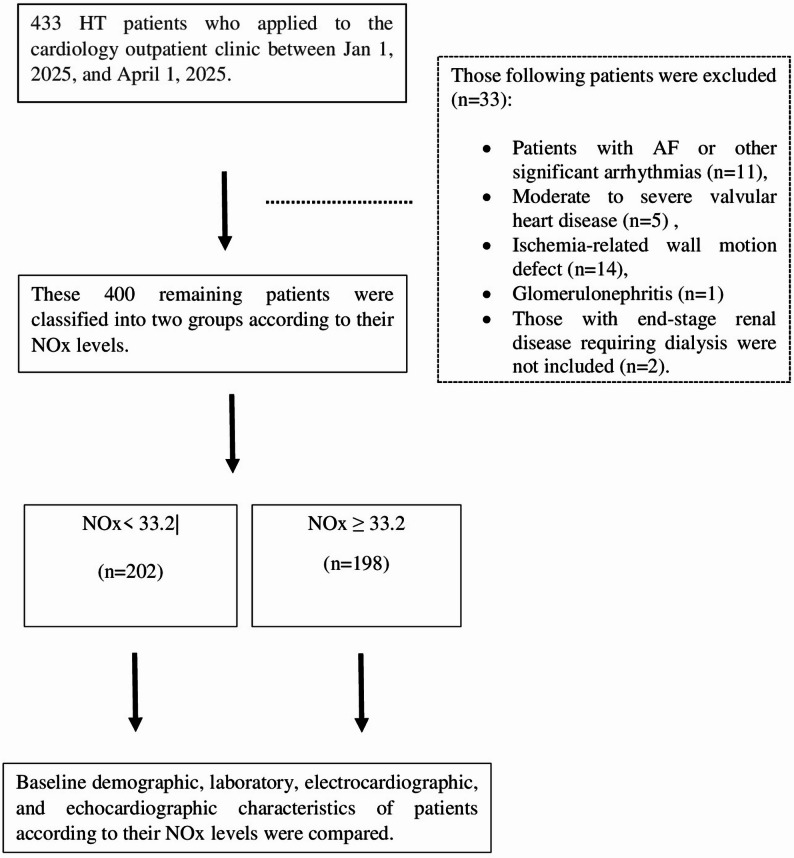
Flowchart illustrating the study design and patient selection process

In this prospectively enrolled cohort with cross-sectional analysis, 433 patients with HT who presented to the cardiology outpatient clinic between January 1 and April 1, 2025, were consecutively screened. Baseline demographic and clinical characteristics were recorded upon admission. All medications were recorded at enrollment. Medications potentially affecting nitric oxide metabolism were also specifically reviewed; among these, only two patients were receiving nitrate therapy, and none were using phosphodiesterase-5 inhibitors. A total of 33 patients were excluded due to conditions that could potentially affect echocardiographic measurements or confound the evaluation of hypertension-related cardiac and renal alterations. These conditions included glomerulonephritis, end-stage renal disease requiring dialysis, ischemia-related wall motion abnormalities, moderate to severe valvular heart disease, and atrial fibrillation or other significant arrhythmias. The remaining 400 patients underwent venous blood and spot urine sampling, followed by comprehensive transthoracic echocardiographic evaluation (Fig. 1).

The study protocol was approved by the local ethics committee (Ethics Committee of the Dean of the Faculty of Medicine of Kafkas University, approval number 80576354-050-99/617) and adhered to the principles outlined in the Declaration of Helsinki. Written informed consent was obtained from all participants.

### Blood and urine analyses

Complete blood count, renal function tests (blood urea and serum creatinine), and lipid profiles were performed as part of the routine laboratory evaluation. In addition, serum NOx levels and spot UACR were assessed. A first-morning, midstream, clean-catch spot urine sample was collected after overnight fasting; albumin was measured by immunoturbidimetry and creatinine by an enzymatic method, and UACR was expressed as mg/g. Urine samples were processed and analyzed on the same day of collection without freezing. In accordance with the 2024 ESC Guidelines for the management of elevated blood pressure and hypertension, albuminuria was defined as UACR ≥ 30 mg/g (≥ 3 mg/mmol) [13]. Venous blood was drawn after overnight fasting into serum separator tubes, centrifuged at 4000 g for 10 min at 4 °C, and serum aliquots were stored at − 25 °C until analysis (single freeze–thaw). Serum NOx levels were quantified by the modified Griess method [14]: nitrate was reduced to nitrite with VCl₃, total nitrite reacted with sulfanilamide and N-(1-naphthyl) ethylenediamine dihydrochloride in acidic medium to form an azo dye, and absorbance was read at 540 nm on a microplate spectrophotometer. Results were reported as µmol/L NOx; all samples were run in duplicate with internal quality control. Short-term dietary nitrate intake, oral antibacterial use affecting nitrate–nitrite metabolism, and renal clearance–related effects, despite the exclusion of patients with end-stage renal disease requiring dialysis, could not be fully standardized and were considered potential sources of residual confounding. The intra-assay and inter-assay coefficients of variation for the NOx assay were < 8% and < 10%, respectively, and the lower limit of detection was approximately 0.5 µmol/L nitrite. Serum endothelial (eNOS) and inducible (iNOS) nitric oxide synthase concentrations were measured using sandwich ELISA according to the manufacturers’ instructions; assays were run in duplicate.

### Transthoracic echocardiography examination

Imaging was performed using a Philips EPIQ 7 C ultrasound system (Philips Healthcare, Andover, MA, USA) equipped with an X5-1 transducer, and all offline analyses were completed with QLAB software in accordance with ASE/EACVI chamber quantification guidelines [15, 16].

Interventricular septal thickness and posterior wall thickness were measured at the end-diastole in the parasternal long-axis view using the leading-edge-to-leading-edge method.

Left ventricular end-diastolic and end-systolic volumes were assessed in apical four- and two-chamber views and used to compute the left ventricular ejection fraction based on Simpson’s biplane method. Left atrial maximal and minimal volumes were measured using the modified biplane Simpson’s method from both apical views.

Early (E) and late (A) transmitral diastolic velocities were measured using pulsed-wave Doppler in the apical four-chamber view, followed by E/A ratio calculation. Tissue Doppler imaging at the septal and lateral mitral annulus provided early (Em) and late (Am) diastolic velocities, and the E/e′ ratio was derived accordingly.

LV-GLS was measured using speckle-tracking echocardiography and postprocessing with QLAB Advanced Quantification software. LV-GLS measurements were performed in a centralized core laboratory by an experienced cardiologist (A.A.), who was blinded to clinical characteristics and laboratory results, using speckle-tracking echocardiography, with manual tracing of the endocardial borders in the 2-, 3-, and 4-chamber views at end-systole. Strain values were automatically generated for each segment. Image acquisition frame rates were maintained within the guideline-recommended range for speckle-tracking echocardiography.

To assess intra-observer reproducibility, LV-GLS measurements were repeated in 30 randomly selected patients. The intra-class correlation coefficient (ICC) showed excellent agreement (ICC = 0.91, 95% CI: 0.88–0.94, *p* < 0.001). Inter-observer variability was not assessed, as all LV-GLS measurements were performed by a single observer.

Impaired LV-GLS was defined as values > − 15.9% (e.g., − 15%, − 14%) [17]. The LV-GLS cut-off value of − 15.9% was adopted based on previously published literature and applied within a single-vendor, single-software setting; therefore, this threshold should be interpreted in the context of the specific imaging platform used.

### Statistical analysis

All statistical analyses were conducted using SPSS software, version 22.0 (SPSS Inc., Chicago, IL, USA). Continuous variables were expressed as mean ± standard deviation (SD) if normally distributed and as median with interquartile range (IQR) if not. Categorical variables are presented as percentages. The Kolmogorov–Smirnov test was used to assess the normality of distribution for continuous variables. Group comparisons were performed using either the independent samples t-test or the Mann–Whitney U test, depending on the data distribution. NOx was modeled as a continuous variable in all regression analyses. Because no validated clinical threshold exists, the cohort median (33.2 µmol/L) was used only for descriptive dichotomisation in Table 1. There were no missing data for the variables included in the main analyses; therefore, no imputation procedures were applied.

**Table 1:** Comparison of demographic, clinical, laboratory, and echocardiographic parameters between patients with NOx < 33.2 and NOx ≥ 33.2

Variable	NOx < 33.2 (*n* = 202)	NOx ≥ 33.2 (*n* = 198)	Total (*n* = 400)	*p*-value
Age (years)	60 ± 12	58 ± 13	59 ± 13	0.072
Female gender, n (%)	112 (55.4)	102 (51.5)	214 (53.5)	0.431
CAD, n (%)	34 (16.8)	31 (15.7)	65 (16.3)	0.750
DM, n (%)	45 (22.3)	31 (15.7)	76 (19.0)	0.092
Smoking, n (%)	46 (22.8)	52 (26.3)	98 (24.5)	0.418
HL, n (%)	57 (28.2)	36 (18.2)	93 (23.3)	**0.018**
BSA (m^2^)	1.95 ± 0.22	1.95 ± 0.20	1.95 ± 0.21	0.732
ASA (%)	50 (24.8)	49 (24.7)	99 (24.8)	0.999
ACEi (%)	40 (19.8)	35 (17.7)	75 (18.8)	0.603
ARB (%)	62 (30.7)	60(30.3)	122 (30.5)	0.959
BB (%)	55 (27.2)	46 (23.2)	101 (25.3)	0.358
MRA (%)	4(2.0)	2(1.0)	6(1.5)	0.429
Thiazide (%)	47 (23.3)	54 (27.3)	101 (25.3)	0.342
DHP-CCB (%)	56 (27.7)	42 (21.2)	98 (24.5)	0.138
Statin (%)	34 (16.8)	36 (18.2)	70 (17.5)	0.723
SBP (mmHg)	139 ± 17	140 ± 17	139 ± 17	0.971
DBP (mmHg)	85 ± 12	87 ± 12	86 ± 12	0.330
Glucose (mg/dL)	111 ± 51	103 ± 38	107 ± 44	0.296
Creatinine (mg/dL)	1.0 ± 0.5	0.9 ± 0.2	1.0 ± 0.4	0.773
eGFR (mL/min/1.73 m²)	82 ± 17	83 ± 16	83 ± 17	0.465
Na (mmol/L)	139 ± 2	139 ± 2	139 ± 2	0.818
K (mmol/L)	4.2 ± 0.4	4.2 ± 0.4	4.2 ± 0.4	0.859
Hb (g/dL)	14.5 ± 1.9	14.8 ± 1.7	14.7 ± 1.8	0.127
PLT (10^3/uL)	234 ± 63	232 ± 71	233 ± 67	0.594
WBC (10^3/uL)	7.3 ± 1.9	7.3 ± 2.0	7.3 ± 1.9	0.558
CRP (mg/L)	3.4 (1.8–7.1)	3.3 (1.3–5.8)	3.3(1.6–6.3)	0.199
TSH (mIU/L)	1.8(1.1–2.6)	1.6(0.9–2.3)	1.6(1-2.5)	0.289
LDL-C (mg/dl)	125 ± 41	121 ± 39	123 ± 40	0.294
HDL-C (mg/dl)	50 ± 11	50 ± 12	50 ± 12	0.648
HbA1c (%)	6.2 ± 1.4	5.9 ± 0.9	6.1 ± 1.2	**0.002**
BNP (ng/L)	36(18–63)	33(16–68)	34(17–64)	0.768
UACR (mg/g)	50(9-123)	16(9–34)	22(9-115)	**< 0.001**
NOx µmol/L	26.5 ± 5.4	38.1 ± 3.2	32.2 ± 7.3	**< 0.001**
eNOS pg/mL	278 ± 127	284 ± 116	281 ± 122	0.470
iNOS pg/mL	1736 ± 323	1808 ± 363	1771 ± 344	**0.010**
LA-AP (cm)	3.6 ± 0.4	3.6 ± 0.4	3.6 ± 0.4	0.285
IVSd (cm)	1.2 ± 0.2	1.2 ± 0.3	1.2 ± 0.2	0.155
LVPWd (cm)	1.09 ± 0.17	1.06 ± 0.19	1.07 ± 0.18	0.101
LVESD (cm)	3.22 ± 0.58	3.31 ± 0.52	3.27 ± 0.55	**0.018**
LVEDD (cm)	4.4 ± 0.5	4.4 ± 0.5	4.4 ± 0.5	0.374
E, mitral (cm/s)	66 ± 18	63 ± 16	65 ± 17	0.183
A, mitral (cm/s)	85 ± 19	79 ± 15	82 ± 17	**0.001**
E/A, mitral	0.82 ± 0.30	0.82 ± 0.25	0.82 ± 0.27	0.510
DT, (ms)	213 ± 50	210 ± 51	212 ± 50	0.338
Em lateral, mitral (cm/s)	8.6 ± 3.1	8.9 ± 2.9	8.8 ± 3.0	0.335
Am lateral, mitral (cm/s)	11.7 ± 2.7	11.9 ± 2.7	11.8 ± 2.7	0.843
Em septal, mitral (cm/s)	6.6 ± 2.0	6.8 ± 2.2	6.7 ± 2.1	0.669
Am septal, mitral (cm/s)	10.2 ± 2.3	10.2 ± 2.3	10.2 ± 2.3	0.980
E/Em mean, mitral	8.3 (6.9–10.7)	8.2 (6.7–9.5)	8.3 (6.8–10.2)	**0.040**
LA maximum volume	45 ± 15	46 ± 18	46 ± 17	0.540
LA minimum volume	15 (11–22)	16 (11–22)	15(11–22)	0.779
LVESV (mL)	29 ± 12	30 ± 12	30 ± 12	0.878
LVEDV (mL)	72 ± 21	73 ± 20	73 ± 21	0.425
LVEF, (%)	59 ± 7	59 ± 8	59 ± 8	0.639
LV-GLS, (%)	–13.5 ± 3.9	–19.4 ± 5.8	–16.4 ± 5.7	**< 0.001**

*CAD* coronary artery disease, *DM* diabetes mellitus, *HL* hyperlipidemia, *BSA* body surface area, *ASA* acetylsalicylic acid, *ACEi* angiotensin-converting-enzyme inhibitors, *ARB* angiotensin receptor blocker, *BB* beta blocker, *MRA* mineralocorticoid receptor antagonist, *DHP-CCB* dihydropyridine calcium channel blocker, *SBP* systolic blood pressure, *DBP* diastolic blood pressure, *eGFR* estimated glomerular filtration rate, *WBC* white blood cell, *CRP* C-reactive protein, *TSH* thyroid-stimulating hormone, *LDL-C* low-density lipoprotein cholesterol, *HDL-C* high-density lipoprotein cholesterol, *BNP* brain natriuretic peptide, *UACR* urine albumin/creatinine ratio, *NOx* nitrite + nitrate, *eNOS* endothelial nitric oxide synthase, *iNOS* inducible nitric oxide synthase, *LA-AP* left atrial anterior-posterior diameter, *IVSd* inter-ventricular septal thickness in diastole, *LVPWd* left ventricular posterior wall thickness at end of diastole, *LVESD* left ventricular end-systolic diameter, *LVEDD* left ventricular end-diastolic diameter, *E* peak early inflow velocity, *A* peak late inflow velocity, *DT* deceleration time, *Em* early diastolic mitral annular tissue Doppler velocity, *Am* late diastolic mitral annular tissue Doppler velocity, *LVESV* left ventricular end-systolic volume, *LVEDV* left ventricular end-diastolic volume, *LVEF* left ventricle ejection fraction, *LV-GLS* left ventricular global longitudinal strain

Bold values indicate statistically significant results (*p* < 0.05)

Separate multivariable logistic regression models were constructed for impaired LV-GLS and for albuminuria. For each outcome, all variables were initially evaluated using univariate logistic regression analyses, and variables showing a statistically significant association at *p* < 0.05 were considered candidates for multivariable model construction. In addition to variables significant in univariate analyses, estimated glomerular filtration rate (eGFR) was retained in the multivariable models because of its known physiological relevance to NOx metabolism and renal clearance. Furthermore, pre-specified clinical core models were constructed separately for each outcome to assess the robustness of the associations. For impaired LV-GLS, the clinical core model included key clinical confounders selected a priori based on clinical relevance, including age, gender, smoking status, eGFR, HbA1c, BNP, UACR, NOx, mitral E/A ratio, systolic blood pressure (SBP), use of angiotensin-converting enzyme inhibitors (ACEi) or angiotensin receptor blockers (ARBs), and statin therapy. For albuminuria, the clinical core model included age, gender, smoking status, eGFR, HbA1c, BNP, NOx, SBP, use of ACEi or ARBs, statin therapy, and LV-GLS. Multivariable models were built using a backward conditional selection method. Multicollinearity among variables included in each final model was assessed using the Variance Inflation Factor (VIF), and no significant multicollinearity was detected.

For each independent variable, odds ratios (ORs) and 95% confidence intervals (CIs) were calculated. Receiver operating characteristic (ROC) curve analyses were subsequently performed to evaluate the discriminative ability of NOx for impaired LV-GLS and albuminuria. Outcome-specific optimal cut-off values were determined using the Youden index. Diagnostic performance was quantified by calculating the area under the curve (AUC).

Model calibration was assessed using the Brier score, which reflects the mean squared difference between predicted probabilities and observed outcomes.

To internally validate the robustness of the regression coefficients and identified cut-off values, bootstrap resampling was performed. Multivariable logistic regression models were subjected to bootstrap validation using 1,000 resamples. Bias-corrected estimates, standard errors, and 95% confidence intervals were calculated for regression coefficients to assess model stability. A p-value < 0.05 was considered statistically significant throughout the study.

## Results

In this study, the mean age of the 400 patients was 59 ± 13 years, and 53.5% of them were female. Albuminuria was present in 169 patients (42.3%), while impaired left ventricular global longitudinal strain (LV-GLS) was observed in 204 patients (51%). Based on the median of NOx, patients were divided into these two groups: NOx < 33.2 (*n* = 202, 50.5%) and NOx ≥ 33.2 (*n* = 198, 49.5%). The NOx ≥ 33.2 group had a significantly lower UACR (16 [9–34] vs. 50 [9–123]; *p* < 0.001), A, mitral (79 ± 15 cm/s vs. 85 ± 19 cm/s; *p* = 0.001), E/Em mean mitral (8.2 [6.7–9.5] vs. 8.3 [6.9–10.7]; *p* = 0.040), and HbA1c value (5.9 ± 0.9 vs. 6.2 ± 1.4; *p* = 0.010) than the NOx < 33.2 group. In addition, iNOS levels (1808 ± 363 vs. 1736 ± 323; *p* = 0.010) and LV-GLS (–19.4 ± 5.8 vs. − 13.5 ± 3.9; *p* < 0.001) were significantly higher in patients with NOx ≥ 33.2. Table 1 displays the patients’ baseline characteristics and their echocardiographic and laboratory values.

In the primary multivariable model for impaired LV-GLS (Table 2), age, UACR, E/A ratio, and NOx (OR = 0.805; 95% CI: 0.765–0.847; *p* < 0.001) were independently associated with impaired LV-GLS. To further evaluate the robustness of these associations, a pre-specified clinical core model was constructed, including established confounders (Table 3). In this model, age, smoking status, UACR, E/A ratio, and NOx were independently associated with impaired LV-GLS. For albuminuria, the results were consistent across both the primary and clinical core models (Tables 4 and 5), with NOx (OR = 0.902; 95% CI: 0.872–0.933; *p* < 0.001) and BNP identified as independent predictors.

**Table 2:** Univariate and multivariate logistic regression analyses of factors associated with impaired LV-GLS

Variable	Univariate			Multivariate		
	Odds ratio	95% CI	*p*-value	Odds ratio	95% CI	*p*-value
Age (years)	1.026	1.010–1.043	0.002	1.025	1.002–1.048	**0.036**
eGFR (mL/min/1.73 m²)	0.989	0.978–1.001	0.079			
HbA1c, %	1.499	1.170–1.921	0.001			
BNP ng/L	1.003	1.000-1.006	0.045			
UACR mg/g	1.002	1.001–1.004	0.002	1.002	1.000-1.003	**0.035**
NOx µmol/L	0.817	0.783–0.853	< 0.001	0.812	0.773–0.852	**< 0.001**
E/A, mitral	0.253	0.108–0.593	0.002	0.251	0.083–0.765	**0.015**

*LV-GLS* left ventricular global longitudinal strain, *eGFR* estimated glomerular filtration rate, *HbA1c* hemoglobin A1c, *BNP* brain natriuretic peptide, *UACR* urine albumin-to-creatinine ratio, *NOx*: nitrite + nitrate, *E/A*,* mitral* ratio of early to late mitral inflow velocities

Bold values indicate statistically significant results (*p* < 0.05) in multivariate analysis

**Table 3:** Univariate and multivariate logistic regression analyses of the clinical core model for impaired LV-GLS

Variable	Univariate			Multivariate		
	Odds ratio	95% CI	*p*-value	Odds ratio	95% CI	*p*-value
Age (years)	1.026	1.010–1.043	0.002	1.031	1.006–1.057	**0.014**
Female gender, n (%)	0.856	0.578–1.269	0.439			
Smoking, n (%)	1.240	0.786–1.958	0.355	0.498	0.261–0.951	**0.035**
eGFR (mL/min/1.73 m²)	0.989	0.978–1.001	0.079			
HbA1c, %	1.499	1.170–1.921	0.001			
BNP ng/L	1.003	1.000-1.006	0.045			
UACR mg/g	1.002	1.001–1.004	0.002	1.002	1.000-1.003	**0.019**
NOx µmol/L	0.817	0.783–0.853	< 0.001	0.802	0.762–0.844	**< 0.001**
E/A, mitral	0.253	0.108–0.593	0.002	0.257	0.088–0.750	**0.013**
SBP (mmHg)	1.004	0.995–1.014	0.392			
ACEi/ARB (%)	1.502	0.905–2.493	0.116	1.854	0.990–3.470	0.054
Statin (%)	1.293	0.770–2.169	0.331			

*LV-GLS* left ventricular global longitudinal strain, *eGFR* estimated glomerular filtration rate, *HbA1c* hemoglobin A1c, *BNP* brain natriuretic peptide, *UACR* urine albumin-to-creatinine ratio, *NOx* nitrite + nitrate, *E/A*,* mitral* ratio of early to late mitral inflow velocities, *SBP* systolic blood pressure, *ACEi* angiotensin-converting-enzyme inhibitors, *ARB* angiotensin receptor blocker

Bold values indicate statistically significant results (p < 0.05) in multivariate analysis

**Table 4:** Univariate and multivariate logistic regression analyses of factors associated with albuminuria

Variable	Univariate			Multivariate		
	Odds ratio	95% CI	*p*-value	Odds ratio	95% CI	*p*-value
Age, years	1.017	1.001–1.033	0.042			
eGFR (mL/min/1.73 m²)	0.989	0.977–1.001	0.070			
HbA1c, %	1.321	1.064–1.639	0.012			
BNP ng/L	1.003	1.000-1.006	0.029	1.004	1.000-1.007	**0.034**
NOx µmol/L	0.905	0.877–0.934	< 0.001	0.902	0.872–0.932	**< 0.001**
LV-GLS, %	1.060	1.021-1.100	0.002			

*HbA1c* hemoglobin A1c, *BNP* brain natriuretic peptide, *NOx* nitrite + nitrate, *LV-GLS* left ventricular global longitudinal strainBold values indicate statistically significant results (p < 0.05) in multivariate analysis

**Table 5:** Univariate and multivariate logistic regression analyses of the clinical core model for albuminuria

Variable	Univariate			Multivariate		
	Odds ratio	95% CI	*p*-value	Odds ratio	95% CI	*p*-value
Age (years)	1.017	1.001–1.033	**0.042**			
Female gender, n (%)	1.208	0.811-1.800	0.352			
Smoking, n (%)	0.978	0.616–1.552	0.924			
eGFR (mL/min/1.73 m²)	0.989	0.977–1.001	0.070			
HbA1c, %	1.321	1.064–1.639	**0.012**			
BNP ng/L	1.003	1.000-1.006	**0.029**	1.004	1.000-1.007	**0.033**
NOx µmol/L	0.905	0.877–0.934	**< 0.001**	0.902	0.872–0.933	**< 0.001**
SBP (mmHg)	1.007	0.995–1.018	0.255			
ACEi/ARB (%)	1.161	0.700-1.925	0.563			
Statin (%)	0.960	0.569–1.620	0.878			
LV-GLS, %	1.060	1.021-1.100	**0.002**			

*eGFR* estimated glomerular filtration rate, *HbA1c* hemoglobin A1c, *BNP* brain natriuretic peptide, *NOx* nitrite + nitrate, *SBP* systolic blood pressure, *ACEi* angiotensin-converting-enzyme inhibitors, *ARB* angiotensin receptor blocker, *LV-GLS* left ventricular global longitudinal strain

Bold values indicate statistically significant results (p < 0.05)

Model calibration was additionally assessed using the Brier score. The Brier score was 0.164 for the impaired LV-GLS model and 0.231 for the albuminuria model, indicating acceptable overall calibration. After bootstrap validation using 1,000 resamples, the association between NOx and impaired LV-GLS remained statistically significant (bootstrap coefficient B = − 0.207; 95% CI: −0.273 to − 0.165; *p* < 0.001). Similarly, bootstrap analysis using 1,000 resamples confirmed a statistically significant association between NOx and albuminuria (bootstrap coefficient B = − 0.110; 95% CI: −0.161 to − 0.067; *p* < 0.001).

**Fig. 2:**
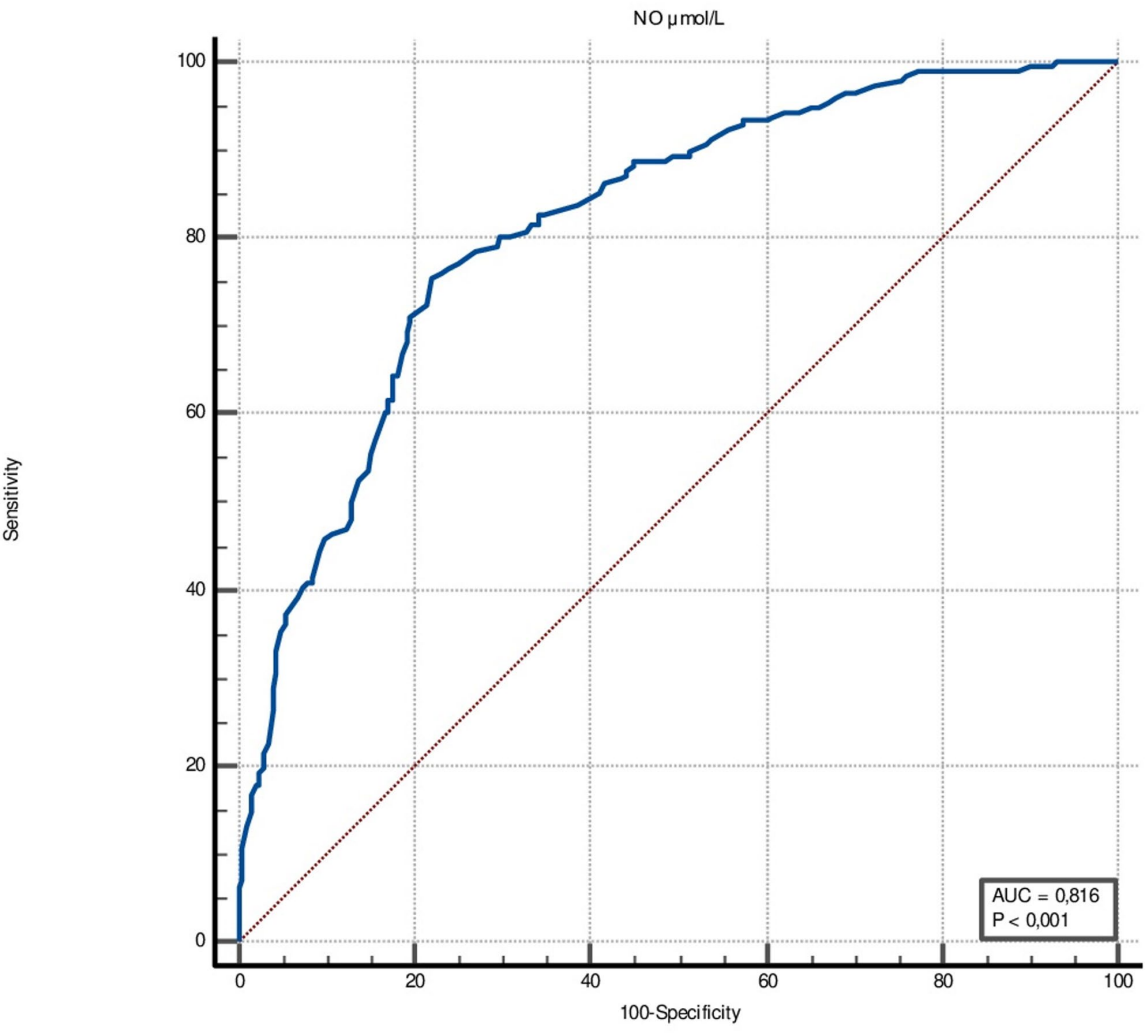
ROC curve of serum NOx levels for impaired LV-GLS. The AUC was 0.816 with a 95% CI of 0.775–0.853 (*p* < 0.001). ROC: Receiver operating characteristic; NOx: nitrite + nitrate; LV-GLS: left ventricular global longitudinal strain; AUC: area under the curve; CI: confidence interval

**Fig. 3:**
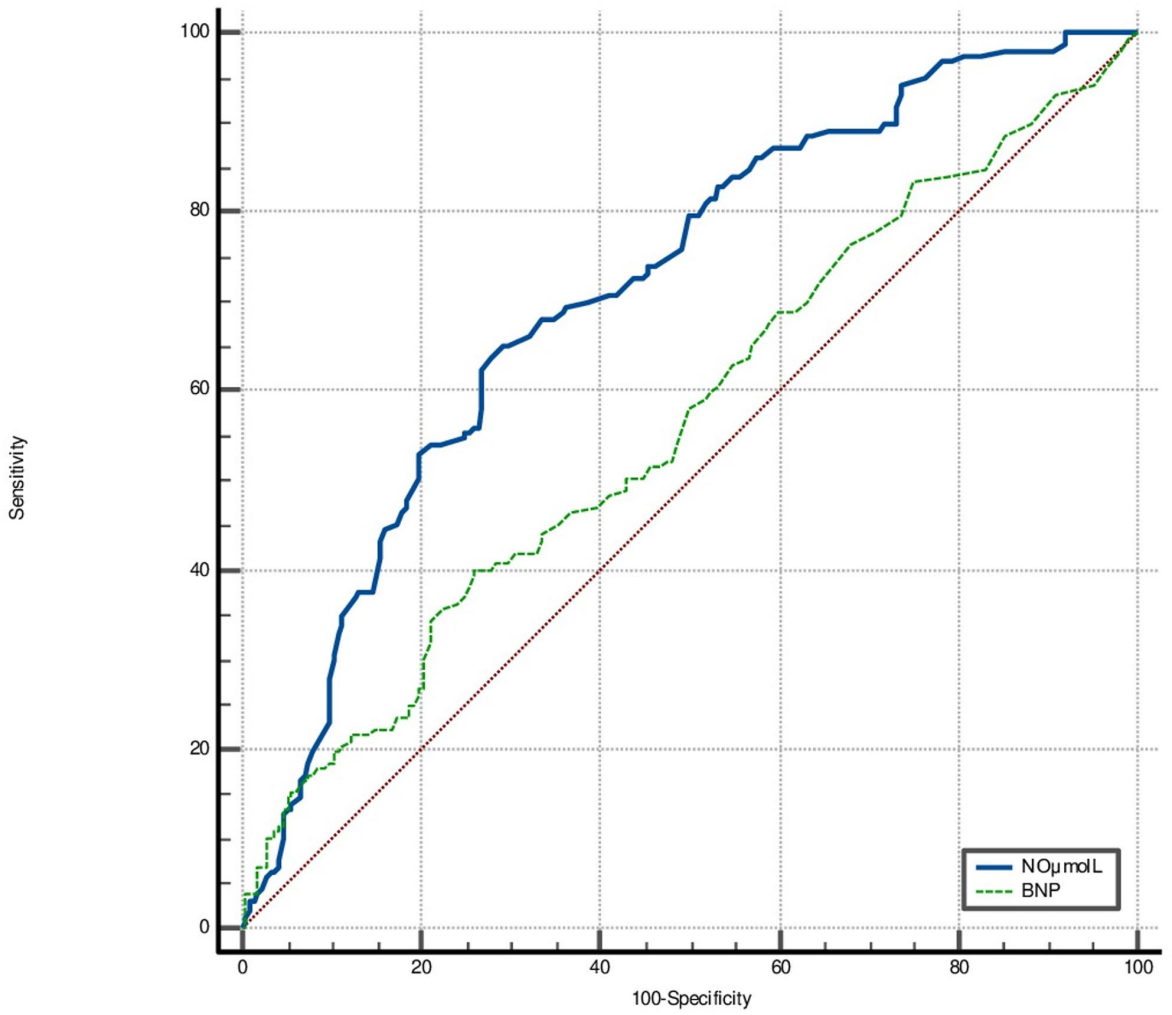
ROC curves comparing serum NOx and BNP levels for albuminuria. AUC was 0.710 (95% CI: 0.663–0.754) for NOx and 0.567 (95% CI: 0.515–0.618) for BNP. ROC: Receiver operating characteristic; NOx: nitrite + nitrate; BNP: B-type natriuretic peptide; AUC: area under the curve

In the ROC curve analysis, the AUC for NOx was 0.816 for impaired LV-GLS (95% CI: 0.775–0.853; *p* < 0.0001) (Fig. 2). With a sensitivity of 75.5% (95% CI: 68.9–81.4) and a specificity of 77.9% (95% CI: 71.6–83.4), a cut-off value for NOx for impaired LV-GLS was lower than 32.8. For albuminuria, NOx demonstrated an AUC of 0.710 (95% CI: 0.663–0.754; *p* < 0.0001), with a sensitivity of 63.9% (95% CI: 56.2–71.1) and specificity of 71.0% (95% CI: 64.7–76.8), and a cut-off value of 32.2 µmol/L. Also, NOx had a higher AUC value than BNP for albuminuria (0.710 vs. 0.567, *p* = 0.0003, respectively) (Fig. 3).

**Fig. 4:**
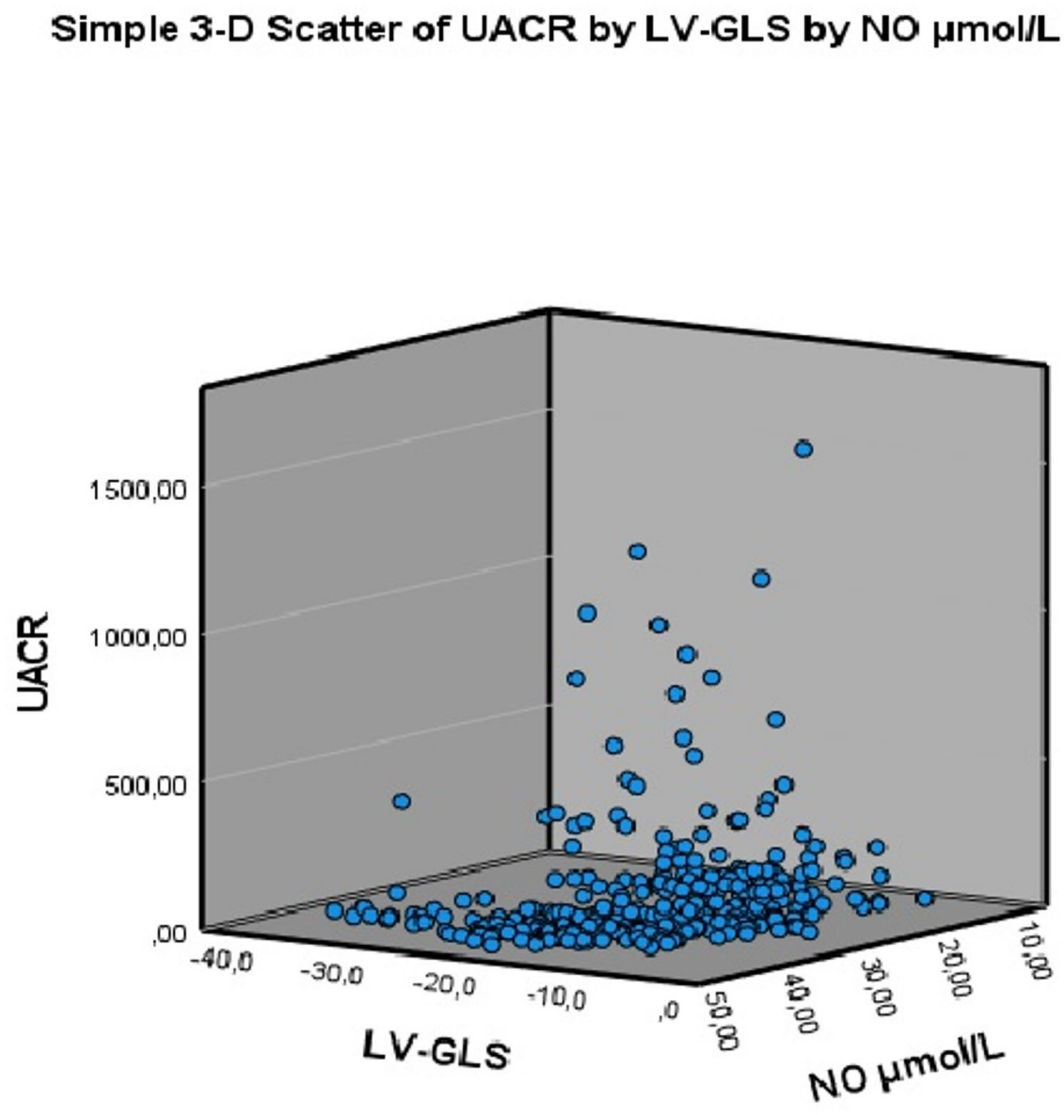
Three-dimensional scatter plot illustrating the relationship between NOx, LV-GLS, and UACR. Each point represents an individual participant. NOx: nitrite + nitrate; LV-GLS: left ventricular global longitudinal strain; UACR: urine albumin/creatinine ratio

At the identified cut-off value, NOx demonstrated a positive predictive value (PPV) of 76.7% (95% CI: 71.5–81.2) and a negative predictive value (NPV) of 76.8% (95% CI: 71.9–81.1) for impaired LV-GLS. For albuminuria, the corresponding PPV and NPV were 61.7% (95% CI: 56.1–67.0) and 72.9% (95% CI: 68.4–77.0), respectively. Finally, the distribution of NOx, LV-GLS, and UACR is illustrated in Fig. 4 using a three-dimensional scatter plot.

## Discussion

In this study, higher NOx levels were associated with better LV-GLS values and lower UACR in hypertensive patients. Multivariate analysis also showed that low NOx levels were independently associated with impaired LV-GLS and increased UACR. Moreover, NOx demonstrated a superior discriminative ability for impaired LV-GLS compared to age and mitral E/Em ratio, as evidenced by the ROC analysis. Additionally, NOx demonstrated a better discriminative ability for albuminuria than that of BNP.

Hypertension is a highly prevalent condition that is associated with morbidity, lower quality of life, and cardiovascular mortality and is associated with increased healthcare costs. In the long term, the worst outcomes of HT mostly arise from HMOD [1]. Various demographic, echocardiographic, and biochemical parameters have been investigated to detect end organ damage in patients with hypertension [18–20]. Among biochemical markers, BNP and C-reactive protein (CRP) have been widely studied due to their relevance to cardiac stress and systemic inflammation [20–22]. The role of NO, however, has not been clearly established regarding HMOD. The role of NO in terms of vascular homeostasis, endothelial function, vasodilation, and oxidative stress balance has already been documented [23, 24]. It has also been shown that NO is associated with HT, renal diseases, and diastolic dysfunction [25–27]. Similarly, in our study, lower NOx levels were associated with higher mitral E/Em mean and A mitral. Further, in the present study, lower NOx levels were associated with impaired LV-GLS and increased UACR in hypertensive patients.

Left ventricular global longitudinal strain is considered one of the most sensitive and reliable echocardiographic tools for detecting structural and functional abnormalities of the left ventricle. It is particularly effective in revealing subtle myocardial damage that occurs in the very early stages of HT before overt clinical or structural changes become apparent [5]. This makes LV-GLS a valuable parameter for the early detection of hypertensive heart disease and for monitoring disease progression in asymptomatic individuals. However, determining LV-GLS is technically demanding: it requires high-frame-rate images, vendor-specific speckle-tracking software, and experienced operators, making it time-consuming and not universally available. Given the technical limitations of strain imaging, researchers have explored alternative, more accessible parameters, including albumin, hemoglobin, eGFR, and BNP [28, 29]. In the present study, we demonstrated for the first time that lower blood NOx levels were independently associated with impaired LV-GLS. The ability of diminished NOx to be associated with strain deterioration may be mechanistically attributed to its central role in endothelial dysfunction. In hypertensive patients, reduced NOx levels contribute to endothelial dysfunction and impaired vascular relaxation [30]. As a consequence of impaired vasodilation and progressive arterial stiffness, afterload increases and may eventually lead to myocardial dysfunction, particularly diastolic impairment. This mechanism is thought to contribute to early changes in left ventricular strain, even in patients with preserved ejection fraction.

Interestingly, in our study, the group with higher NOx levels not only demonstrated better LV-GLS values but also exhibited elevated iNOS levels. While iNOS is generally known as an inducible enzyme upregulated during inflammation, recent studies have found that it may also be constitutively expressed in various tissues, including the myocardium [31, 32]. This raises the possibility that, under certain conditions, iNOS may contribute to physiological NO production rather than reflecting only pathological processes [33]. The concurrent elevation of NOx and iNOS in patients with preserved LV strain may reflect an adaptive or compensatory response of NO-related pathways in early-stage hypertensive heart disease. Nevertheless, this observation should be interpreted with caution. The association between higher NOx and iNOS levels and preserved LV strain should not be interpreted as evidence of a direct protective effect of iNOS itself. Alternatively, residual or unmeasured confounding factors may partly explain this association. Given the cross-sectional design of the present study, causal inferences cannot be drawn, and this finding should be considered hypothesis-generating, warranting confirmation in future longitudinal and mechanistic studies.

Renal injury is among the best-known HMOD. Kidney involvement is typically assessed and standardized by monitoring the UACR, which serves as an early and sensitive marker of glomerular dysfunction. Considering this feature of UACR, to date, several parameters have been introduced that are associated with renal impairment and UACR [34]. In our study, reduced NOx was independently associated with albuminuria. This association may be explained by the key role of endothelial dysfunction in the development of albuminuria. Decreased NOx levels impair vasodilation, increase glomerular capillary pressure, and promote endothelial dysfunction, ultimately enhancing the permeability of the glomerular filtration barrier and facilitating albumin leakage into the urine. These pathophysiological effects have been further verified by experimental studies showing that NO promotes renal vasodilation and modulates glomerular hemodynamics [35, 36].

In hypertensive patients, a comprehensive evaluation of all forms of HMOD in the long-term process is often challenging. Given the association of organ damage with reduced quality of life, increased healthcare costs, and elevated cardiovascular mortality, the use of a practical, accessible marker to assess such damage appears both reasonable and clinically valuable. In this context, NOx emerges as a promising candidate. It can be easily obtained from a single blood sample and may offer simultaneous insight into both myocardial and renal functions. In our study, NOx showed a stronger association with impaired LV-GLS than age or mitral E/Em ratio. These findings support the potential role of NOx as a practical and integrative biochemical parameter for the early identification of subclinical target organ damage in HT.

The use of NO as a single integrative parameter may offer an opportune approach for early identification of HMOD. Since organ damage in HT is typically progressive and often asymptomatic in its early stages, recognizing high-risk individuals before irreversible damage occurs may be crucial. In this context, NOx may serve as a practical and informative marker of the need for further functional evaluation.

There are a few limitations in this study to acknowledge. First, it was conducted in a single center, which may limit the generalizability of the findings to larger hypertensive populations. Second, due to the cross-sectional nature of our analysis, it is difficult to establish a causal relationship between NOx levels and HMOD. That is, determining which is the cause and which is the result cannot be verified. Third, nitric oxide was not measured directly: the modified Griess assay quantifies NOx as an indirect index of NO pathways and cannot distinguish endogenous NO production from dietary nitrate intake or fully capture NO bioactivity. Although samples were collected after overnight fasting and patients with end-stage renal disease requiring dialysis were excluded, short-term dietary nitrate intake, oral antibacterial use affecting nitrate–nitrite metabolism, and residual confounding related to renal clearance could not be fully controlled. In addition, the ROC-derived cut-off values lack external validation and should therefore be interpreted as exploratory and cohort-specific, requiring confirmation in independent populations before any potential clinical application. Finally, although the study focused on HMOD, which is known to be associated with adverse clinical outcomes, longitudinal data on patient survival and prognostic endpoints were not available; thus, future longitudinal studies are warranted to confirm these findings and to clarify their prognostic implications.

## Conclusion

Hypertension is a chronic condition closely linked to progressive HMOD. In clinical practice, evaluating the functional status of each affected organ system individually can be both time consuming and technically demanding. Considering that NOx is easily measurable, widely accessible, and reproducible, it may represent an independent marker for identifying both myocardial dysfunction and renal impairment in hypertensive patients.

## Supplementary Information

Supplementary Material 1.

## Data Availability

The datasets generated and/or analysed during the current study are not publicly available due to institutional and ethical restrictions, but are available from the corresponding author on reasonable request.

## Abbreviations

HT: Hypertension
HMOD: Hypertension-mediated organ damage
LVH: Left ventricular hypertrophy
LV-GLS: left ventricular global longitudinal strain
NO: Nitric oxide
NOx: Circulating nitrite and nitrate
UACR: Urine albumin-to-creatinine ratio
eNOS: Serum endothelial nitric oxide synthase
iNOS: Serum inducible nitric oxide synthase
IQR: Interquartile range
ROC: Receiver operating characteristic
AUC: Area under the curve
