# Supplementary material for: The role of nitric oxide in hypertensive target organ damage in patients without renal impairment: insights from left ventricular global longitudinal strain and albuminuria
Source: BMC Cardiovasc Disord. 2026 Feb 6;26:216. doi: 10.1186/s12872-026-05575-5 (PMC12977595; doi:10.1186/s12872-026-05575-5)
Supplement: Supplementary file 1 — Supplementary Material 1. [file 12872_2026_5575_MOESM1_ESM.docx]

**Supplementary Results**

In exploratory analyses, NOx levels showed a weak but statistically significant positive correlation with iNOS concentrations (Spearman’s rho = 0.148, p = 0.003). In contrast, no significant correlation was observed between NOx and eNOS levels (Spearman’s rho = 0.051, p = 0.309).

These analyses were performed to provide additional mechanistic context and are interpreted in a hypothesis-generating manner.


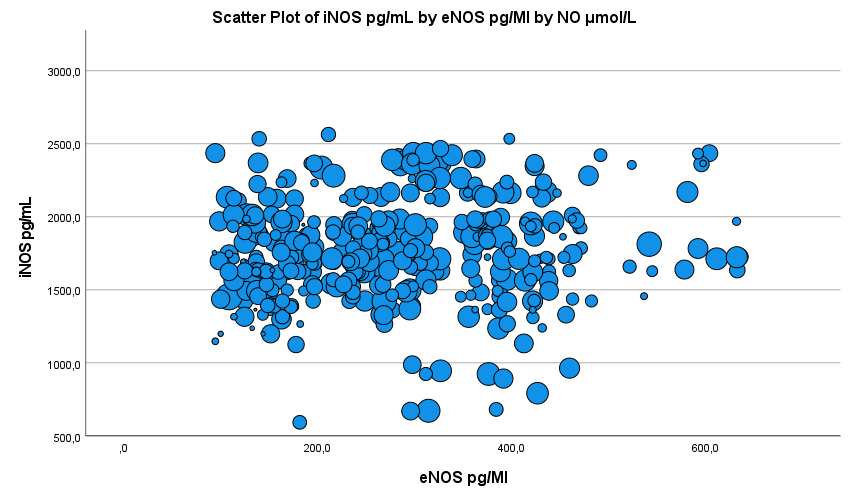


**Supplementary Figure 1.** Scatter plot illustrating the relationship between NOx and nitric oxide synthase isoforms (iNOS and eNOS). Each point represents an individual participant. This figure is provided as an exploratory visualization to support the supplementary analyses examining potential mechanistic associations between NOx and NOS isoforms.

NOx: nitrite + nitrate; eNOS: endothelial nitric oxide synthase; iNOS: inducible nitric oxide synthase.
